# Enhancing the Generated Stable Correlation in a Dissipative System of Two Coupled Qubits inside a Coherent Cavity via Their Dipole-Dipole Interplay

**DOI:** 10.3390/e21070672

**Published:** 2019-07-09

**Authors:** Abdel-Baset A. Mohamed, Mostafa Hashem, Hichem Eleuch

**Affiliations:** 1 Department of Mathematics, College of Science and Humanities in Al-Aflaj 11942, Prince Sattam Bin Abdulaziz University, Al-Kharj 11942, Saudi Arabia; 2 Faculty of Science, Assiut University, Assiut 71515, Egypt; 3 Department of Applied Sciences and Mathematics, College of Arts and Sciences, Abu Dhabi University, Abu Dhabi 59911, UAE; 4 Institute for Quantum Science and Engineering, Texas A&M University, College Station, TX 77843, USA

**Keywords:** mon-classical correlations, dipole-dipole interplay, intrinsic dissipation, 0 3. 65. Yz, 03. 65. Ud, 42. 50. Lc

## Abstract

We explore the dissipative dynamics of two coupled qubits placed inside a coherent cavity-field under dipole-dipole interplay and 2-photon transitions. The generated non-classical correlations (NCCs) beyond entanglement are investigated via two measures based on the Hilbert-Schmidt norm. It is found that the robustness of the generated NCCs can be greatly enhanced by performing the intrinsic dissipation rate, dipole-dipole interplay rate, initial coherence intensity and the degree of the coherent state superpositions. The results show that the intrinsic decoherence stabilize the stationarity of the non-classical correlations while the dipole interplay rate boost them. The non-classical correlations can be frozen at their stationary correlations by increasing the intrinsic dissipation rate. Also NCCs, can be enhanced by increasing the initial coherent intensity.

## 1. Introduction

Non-classical correlations (NCCs) are a physical phenomenon and fundamental concept in quantum theory, they identify the quantum aspects of bipartite as well as multipartite systems. NCC has a critical role in setting the boundary between classical and quantum systems. It represents an important physical resource for quantum communication and processing [1].

In principle, NCCs could be generated in a multipartite system. Numerous investigation demonstrate NCCs in several physical systems ranging from atoms and photons to solide-state materials [2,3,4,5]. There are several types of NCCs beyond QE, such as quantum discord [6,7,8], geometric quantum discord (GQD) [9], measurement-induced disturbance [10], measurement-induced nonlocality (MIN) [11] and quantum steering [12,13,14,15], which are different forms of quantum nonlocality [16,17,18]. Its found that steering effects are strongly related to QE [13]. These types of NCCs can be applied in various branches of quantum engineering, quantum cryptography and quantum information [1,2,17].

It was proven that the QD occurs for some separable mixed quantum states. It is more general than the QE [6,7,9,19]. QD is defined via the discrepancy between total quantum mutual information and the classical correlation. The QD computation for a general quantum non X-matrix is very difficult, therefore other types of NCCs are introduced via dual measures such as: geometric quantum discord (GQD) and measurement-induced nonlocality (MIN) [20,21,22,23,24]. GQD depends on the minimization of the discrepancy between given quantum states and classical states set by different norms as: Hilbert-Schmidt norm [9], Schatten one-norm [25] and Bures norm [26]. While MIN quantifies non-classical correlations beyond the non-local correlations. It represents an aspect of the Hilbert-Schmidt norm minimization [9]. Recently a huge efforts were dedicated to the investigations of NCCs in quantum systems [27,28,29,30,31,32,33,34].

Such phenomena of NCCs without QE has been used to accelerate solving some computational scheme in nounitary quantum computation model [35] and has been confirmed by experiments [36]. In a few words, QE is just a special form of NCCs.

In open quantum systems, the useful quantum correlations between the parts of a dissipative system are destroyed. There are several approaches to study the dissipations in open quantum systems [37,38,39]. They are responsible for the transition between the quantum and classical states. Decoherence decrease the NCCs which may induce failure of the algorithms and various protocols of the quantum processing The intrinsic dissipation was introduced in different models [40,41] and was described by a modified or non-unitary Schrodinger equation.

Various nonclassical properties may emerge from the superpositions of coherent states due to the quantum interference between the coherent components [42,43]. These states were experimentally observed in cavity QED [44] and ion-trap systems [45,46].

The NCC dynamics in intrinsic dissipative models have been broadly studied only for the vacuum or number cavity-field [47,48,49] and for two qubits in X states. As far as we know, the effects of the intrinsic dissipation rate and dipole-dipole interplay have not been yet investigated for a two-qubit system prepared in a non-X state inside a cavity.

Such studies are potentially important for the control of the quantum correlations in coupled qubits describing for example several atoms. It opens the door as a substantial ingredients of growing number of applications in quantum technologies. Thus it is of great interest to find relationships between entanglement and different measures of nonclassical correlations. Despite the complexity of the suggested model, we introduce: (i) an analytical description under intrinsic decoherence for two coupled qubits placed inside a cavity where the cavity field is initially in a superposition of coherent states. (ii) we investigate the robustness of the generated NCCs. Furthermore, we investigate the 2-photon transitions and the intrinsic dissipation rate effects on the NCCs. The geometric quantum discord (GQD), the measurement-induced nonlocality and the concurrence are used as quantifiers.

In Section 2, we present the physical model. In Section 3, the definition of the correlation quantifiers are discussed. In Section 4, we analyze the results of the correlation quantifiers. We end-up by a conclusion.

## 2. Time Evolution of Qubits-Cavity Interaction

Our system is formed by two qubits interacting with a coherent cavity field where the 2-photon transitions, dipole-dipole interplay and intrinsic dissipation are considered. The evolution equation of this system is described by Milburn’s equation [40,41]

(1) $$\begin{array}{ccc} \frac{d\rho (t)}{dt} & = & -i\left[H,\rho \left(t\right)\right]-\frac{\gamma }{2}\left[H,\left[H,\rho \left(t\right)\right]\right], \end{array}$$

where γ represents the intrinsic dissipation parameter. The Hamiltonian *H* of the total system, in the rotating-wave approximation, can be written as

(2) $$\begin{array}{c} \begin{array}{ccc} \hat{H} & = & \omega {\hat{a}}^{†}\hat{a}+\sum_{i=1}^{2}\left\{\frac{\omega }{2}{\hat{\sigma }}_{i}^{z}+\lambda \left({\hat{a}}^{2}\sigma_{i}^{+}+{\hat{a}}^{†2}\sigma_{i}^{-}\right)\right\}\\ & & \;+J(\sigma_{1}^{+}\sigma_{2}^{-}+\sigma_{1}^{-}\sigma_{2}^{+}), \end{array} \end{array}$$

where $\hat{a}\left({\hat{a}}^{†}\right)$ presents the annihilation (creation) operator of the cavity field, ω denotes the qubit and the cavity frequencies whereas λ denotes the coupling constant between the cavity and the qubits. The rasing and lowering operators of the *i*-th qubit are ${\hat{\sigma }}_{i}^{\pm }$. *J* represents the dipole-dipole qubit coupling constant.

Here, we assume that the interaction starts with the uncorrelated state as:

(3) $$\begin{array}{c} \rho \left(0\right)=\rho^{AB}\left(0\right)⊗\rho^{F}\left(0\right), \end{array}$$

where the two qubits are initially uncorrelated in their excited states, that is: $\rho^{AB}\left(0\right)=|1_{A}1_{B}\rangle \langle 1_{A}1_{B}|.$ While the cavity-field is started initially in the superposition coherent state as

(4) $$\begin{array}{ccc} \rho^{F}\left(0\right) & = & \frac{{(|\alpha \rangle +\kappa |-\alpha \rangle )(|\alpha \rangle +\kappa |-\alpha \rangle )}^{†}}{1+\kappa^{2}+2\kappa e^{-2N}}, \end{array}$$

where the coherent state, |α〉, is given by

(5) $$\begin{array}{ccc} |\alpha \rangle & = & e^{-N/2}\sum_{n=1}^{\infty }\frac{N^{\frac{n}{2}}}{\sqrt{n!}}|n\rangle , \end{array}$$

where, α its intensity coherence and $N={\left|\alpha \right|}^{2}$ denotes the mean photon number. The values of the parameter κ: κ=0 and 1 are taken respectively for the coherent state and the even coherent state.

From the Equations (1) and (3), we get

(6) $$\begin{array}{ccc} \hat{\rho }\left(t\right) & = & \sum_{m,n=0}\frac{\left[1+\kappa {\left(-1\right)}^{m}\right]\left[1+\kappa {\left(-1\right)}^{n}\right]}{\left[1+\kappa^{2}+2\kappa e^{-2N}\right]\sqrt{m!n!}}N^{\frac{m+n}{2}}e^{-N}\\ & \times & \{\alpha_{11}{\hat{X}}_{11}+\alpha_{13}{\hat{X}}_{13}+\alpha_{14}{\hat{X}}_{14}+\alpha_{31}{\hat{X}}_{31}+\alpha_{41}{\hat{X}}_{41}\\ & & \;+\alpha_{33}{\hat{X}}_{33}+\alpha_{43}{\hat{X}}_{43}+\alpha_{34}{\hat{X}}_{34}+\alpha_{44}{\hat{X}}_{44}\} \end{array}$$

where $\alpha_{mn}=\zeta_{m1}\zeta_{n1}$, and the coefficients $\zeta_{ij}$ satisfy the condition of the eigenvalue-problem: $\hat{H}|\Psi_{m}^{n}\rangle =E_{m}|\Psi_{m}^{n}\rangle$, $E_{m}$ correspond to the eigenvalues. The density matrices dynamics of the dressed states, ${\hat{X}}_{ij}$, are given by

(7) $$\begin{array}{ccc} \;\;{\hat{X}}_{ij}\;\; & = & D^{mn}\left(t\right)e^{-i\lambda (E_{i}^{m}-E_{j}^{n})t}|\Psi_{i}^{m}\rangle \langle \Psi_{j}^{n}|, \end{array}$$

where $D^{mn}\left(t\right)=e^{-\gamma {\left(E_{i}^{m}-E_{j}^{n}\right)}^{2}t}$ is intrinsic noise term. In the space states $\{|\varpi_{1}\rangle =|1_{A}1_{B},n\rangle ,|\varpi_{2}\rangle =|1_{A}0_{B},n+2\rangle ,|\varpi_{3}\rangle =|0_{A}1_{B},n+2\rangle ,|\varpi_{4}\rangle =|0_{A}0_{B},n+4\rangle \;\}$, the used eigenstates $|\Psi_{i}^{n}\rangle$ of the Hamiltonian (1) are given by:

(8) $$\begin{array}{ccc} |\Psi_{m}^{n}\rangle & = & \sum_{k=1}^{4}\zeta_{mk}|\varpi_{k}\rangle ,\left(m=1,2,3,4\right), \end{array}$$

and the corresponding eigenvalues are

(9) $$\begin{array}{c} \begin{array}{ccc} E_{1}^{n} & = & \omega \left(n+1\right)\;E_{2}^{n}=\omega \left(n+1\right)-J,\\ E_{3(4)}^{n} & = & \omega \left(n+1\right)+\frac{1}{2}J\mp \frac{1}{2}\sqrt{J^{2}+8\lambda^{2}\left(\nu_{1}^{n}+\nu_{2}^{n}\right)},\\ \nu_{1}^{n} & = & \frac{(n+2)!}{n!},\;\nu_{2}^{n}=\frac{(n+4)!}{(n+2)!}. \end{array} \end{array}$$

## 3. NCC Quantifiers

To investigate the NCCs of the two qubits via different quantifiers, we need the time evolution for their reduced density matrix, $\rho^{AB}\left(t\right)$, by tracing the cavity-field degrees of freedom as:

(10) $$\begin{array}{c} \rho^{AB}\left(t\right)={tr}_{R}\left\{\rho \left(t\right)\right\}=\sum_{k=0}^{\infty }\langle k|\rho \left(t\right)|k\rangle . \end{array}$$

Now, we can determine the time evolution of NCC quantifiers of GQD and MIN and compare them with the concurrence entanglement.

$\rho^{AB}$, in the Bloch representation using Pauli spin matrices $\sigma_{i}$, can be expressed as:

(11) $$\begin{array}{c} \begin{array}{c} \rho^{AB}=\frac{1}{4}[I_{4\times 4}+\sum_{i=1}\left(x_{i}\sigma_{i}⊗I_{2\times 2}+I_{2\times 2}⊗y_{i}\sigma_{i}\right)\\ \;\;+\sum_{ij=1}r_{ij}\sigma_{i}⊗\sigma_{j}], \end{array} \end{array}$$

where $x_{i}$ and $y_{i}$ are the local Bloch-vectors components, $\vec{x}$ and $\vec{y}$ respectively. While $r_{ij}=tr\left\{\rho^{AB}\left(\sigma_{i}⊗\sigma_{j}\right)\right\}$ are the components of the matrix $R=[r_{ij}]$ [10]. If $\rho_{ij}=\langle i|\rho^{AB}|j\rangle =a_{ij}+ib_{ij}\;\left(i,j=1-4\right)$ are the elements of $\rho^{AB}$, then the vector $\vec{x}$ is

(12) $$\begin{array}{ccc} \vec{x} & = & {\left(2a_{13}+2a_{24},\;2b_{31}+2b_{42},\;2\rho_{11}+2\rho_{22}-1\right)}^{t}, \end{array}$$

and

(13) $$\begin{array}{c} \;\;\;\;\;\;\;\;\;\;\;\;\;\;\;\;\;\;R=2\left(\begin{array}{ccc} a_{23}+a_{14} & b_{23}-b_{14} & a_{13}-a_{24}\\ b_{41}-b_{23} & a_{23}-a_{14} & b_{13}+b_{24}\\ a_{12}-a_{34} & b_{34}-b_{12} & \rho_{11}+\rho_{44}-\frac{1}{2} \end{array}\right). \end{array}$$

**(i) GQD:**

GQD depends on the minimal Hilbert-Schmidt distance between the classical states and the given states [10]. For a general matrix $\rho^{AB}\left(t\right)$, GQD can be written as

(14) $$\begin{array}{ccc} G(t) & = & \frac{1}{4}(∥\vec{x}{∥}^{2}+{∥R∥}^{2}-k_{max}), \end{array}$$

where $k_{max}$ is the largest eigenvalue of the matrix $K=\vec{x}{\vec{x}}^{t}+RR^{t}$.

**(ii) MIN:**

The MIN is a type of NCCs based on the local von Neumann measurements from which one of the quantum reduced states is left invariant [11]. For a general density matrix $\rho^{AB}\left(t\right)$, the expression of MIN is

(15) $$\begin{array}{ccc} M(t) & = & \left\{\begin{array}{cc} \frac{1}{2}(trRR^{t}-\frac{1}{∥x∥}x^{t}RR^{t}x), & x\neq 0;\\ \frac{1}{2}\left(trRR^{t}-\lambda_{min}\right), & x=0. \end{array}\right. \end{array}$$

$\lambda_{min}$ represents the minimum eigenvalue of $RR^{t}$.

**(iii) Concurrence:**

The above functions will be compared with the concurrence entanglement of $\rho^{AB}\left(t\right)$, which is defined as: $C\left(t\right)=\max\left\{0,\sqrt{\lambda_{1}}-\sqrt{\lambda_{2}}-\sqrt{\lambda_{3}}-\sqrt{\lambda_{4}}\;\right\}$, where the quantities $\lambda_{1}>\lambda_{2}>\lambda_{3}>\lambda_{4}$ are the square roots of the eigenvalues of the matrix: $R=\rho^{AB}\left(\sigma_{y}⊗\sigma_{y}\right)\rho^{AB*}\left(\sigma_{y}⊗\sigma_{y}\right)$.

## 4. Dynamics of the Correlation Quantifiers

By using Equation (10) in NCC functions (G(t), M(t) and C(t)), the robustness of the generated NCCs between the two qubits is shown against the intrinsic dissipation rate. We assumed that the two-qubit system is initially uncorrelated.

**Figure 1:**
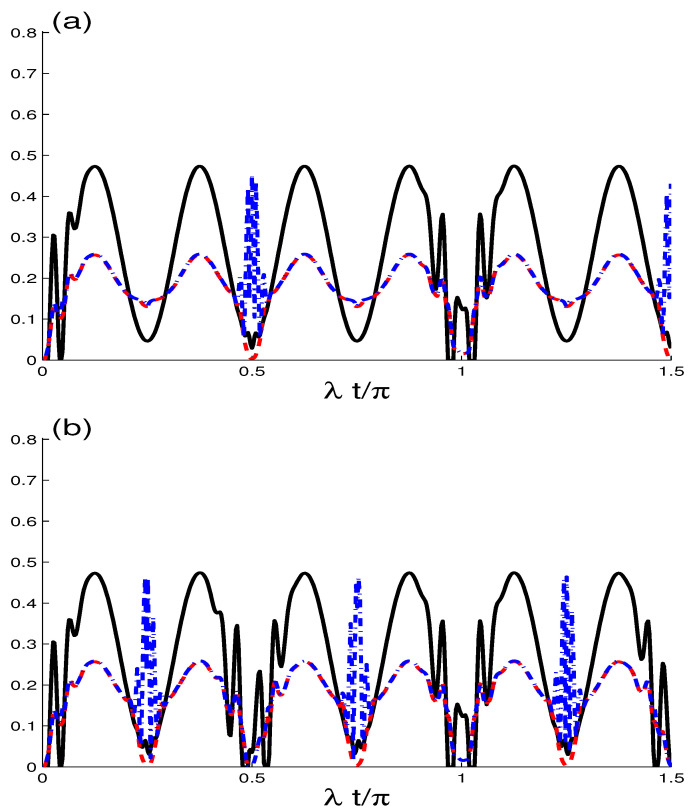
The functions G(t) (dashed curves), M(t) (dot-dashed curves), C(t) (solid curves) as a function of the scaled time λt when N=25, J/λ=0.0 and γ=0.0. For κ=0 in (**a**) and even κ=1 in (**b**).

**Figure 2:**
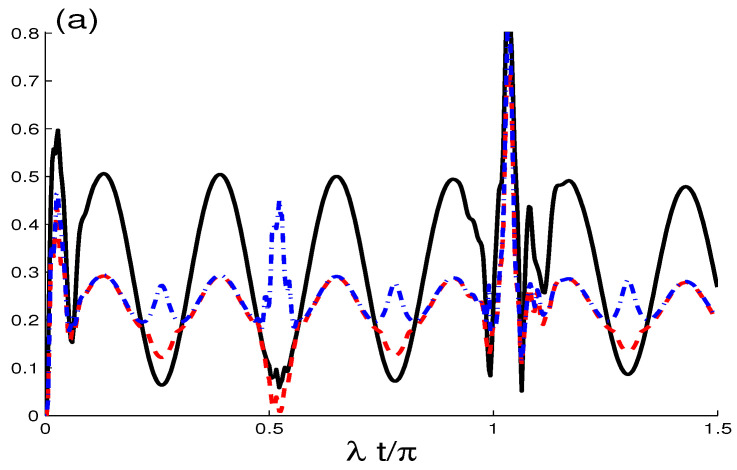

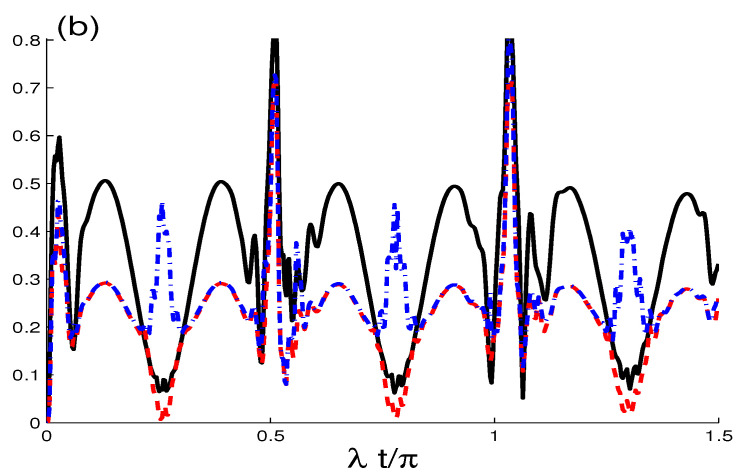
As Figure 1a, but with J/λ=30.

To explore the effect of the superposition parameter κ, we plot the time evolutions of GQD, MIN and concurrence entanglement (see Figure 1a,b) with the initial coherence intensity N=25 and the intrinsic dissipation rate γ=0.0. In Figure 1 and Figure 2 we consider the coherent state κ=0 and J/λ=0.0, we observe that the concurrence grows from zero to its maximal value generating maximal correlated two-qubit states. After that it oscillates with a period nπ, (n=0, 1, 2,…), and the qubits at the end each period are completely disentangled. While at $\lambda t=\left(2n-1\right)\frac{\pi }{8}$ (n=1,2,3,…), C(t) qubits are strongly entangled. Phenomena of sudden growth and death of the concurrence entanglement [50,51,52] are repeated periodically.

On the other hand, the functions of GQD and MIN showing NCC beyond the concurrence entanglement, they have also regular oscillations with a period nπ. We note that G(t), M(t) have the same values, G(t)=M(t), except for small intervals around $\lambda t=\frac{n\pi }{2}$ (n=0,2,3,…). The case of G(t)=M(t) means that the maximization and minimization of Neumann measurements are equal) this type of correlation is known as “*HSD-correlation*”. During the smaller intervals of G(t)≠M(t), the two-qubit states have completely different behavior, we observe at the same time zero-GQD and maximal MIN.

The effect of the superposition coherent parameter κ=1 appears in Figure 1b, where the NCC functions evolve faster than of the case κ=1 and the period reduces to $\frac{n\pi }{2}$, i.e., the dynamical behavior of NCCs depends on the parameter κ. This effect of the superposition parameter is clearly observable on the amplitudes of NCC functions. QE is hastening the death and anabiosis due to the the superposition coherent parameter κ=1 (see Figure 1b).

In Figure 2a,b, the functions G(t), M(t) and C(t) show the dependence of NCCs on the dipole-dipole interplay rate J/λ. Note that there is a significant increase in the number of oscillations as well as the number of peaks. There is also an increase in the extreme values of NCC functions during the interaction period between the qubits. It is clear that dipole-dipole interplay rate inhibits the loss of generated NCCs. Under the influence of the dipole-dipole interaction, the intervals of HSD-correlation appears and the phenomena of sudden growth and death of the entanglement disappears completely.

**Figure 3:**
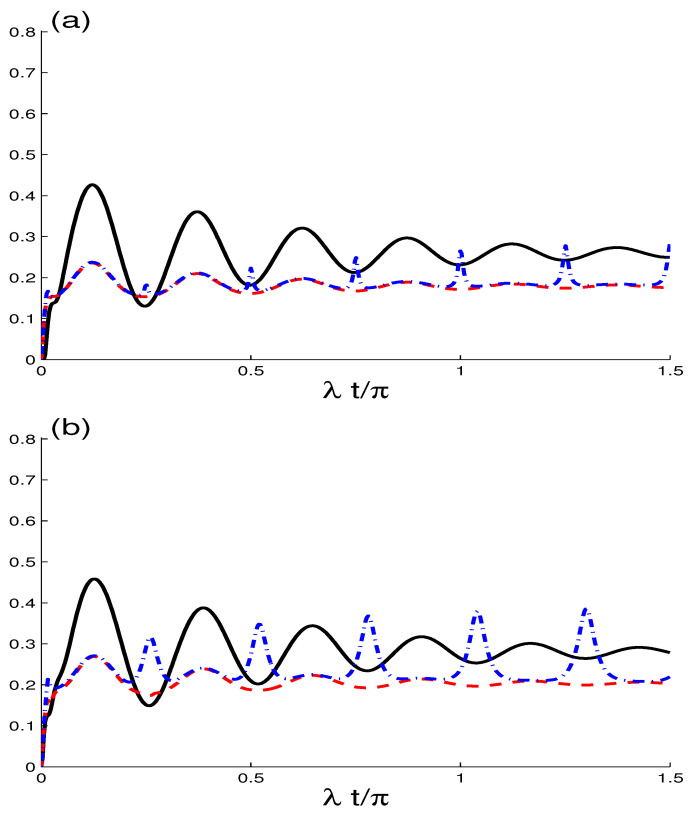
As Figure 1a, but with (J,γ)=(0,0.01)λ in (**a**) and (J,γ)=(30,0.01)λ in (**b**).

In Figure 3a,b is shown the robustness of the generated GQD, MIN and QE, against the intrinsic dissipation rate (γ=0.01λ), when the cavity-field is started initially in two different cases of the coherent-state superposition, κ=0 in Figure 3a and κ=0 in Figure 3b. In Figure 3a, when (J,γ)=(0,0.01)λ (see Figure 3a, we note that GQD, MIN and QE present the same behavior, but they have different amplitudes. With γ=0.01λ, the NCC oscillations are damped and their functions tending to their stable correlation. As time progresses G(t) and C(t) stabilize to their stationary values, while the MIN curve differs from G(t) with smaller peaks. Therefore, the collapses and revivals phenomena are very sensitive for the intrinsic dissipation rate. In general, the stationary HSD-correlation of G(t) and M(t) is always smaller than the stationary concurrence entanglement. We can deduce that the intrinsic dissipation rate leads to the non vanishing stationary correlation of the two-qubit states, i.e., the NCCs are protected by the non-zero intrinsic dissipation rate.

In Figure 3b, the effects of both the intrinsic dissipation and the dipole-dipole interplay are combined by taking (J,γ)=(30,0.01)λ. We note that the collapses and revivals phenomena return to appear in the presence of non-zero intrinsic dissipation rate. Therefore, the dipole-dipole interplay rate leads to delay the stability of generated correlation in the dissipative two-qubit system.

**Figure 4:**
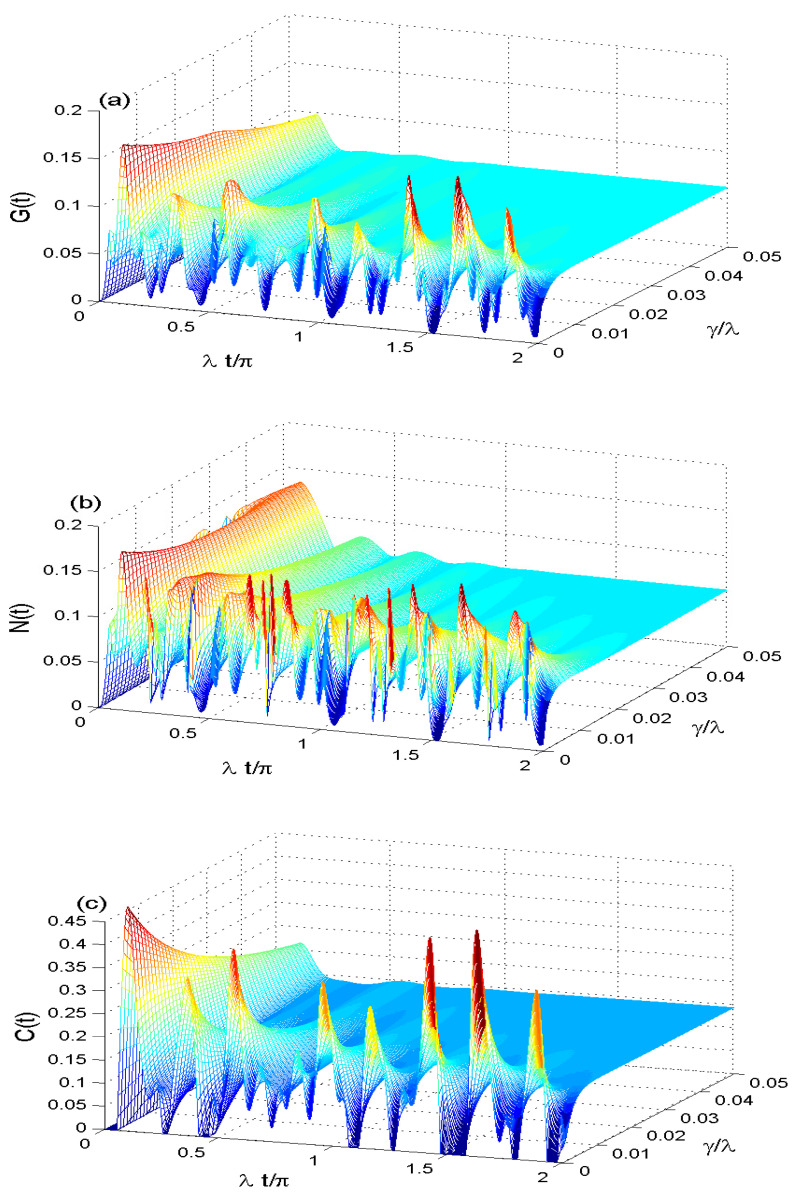
The functions of G(t) in (**a**), M(t) in (**b**) and C(t) in (**c**) when γ/λ∈[0,05] with smaller coherent intensity N=4, J/λ=0.0 and κ=1.

In Figure 4a–c, the functions of MIN, GQD, and QE are plotted as a functions of the scaled time λt and the intrinsic dissipation rate γ/λ∈[0,05] with a smaller initial coherence intensity N=4 and superposition parameter κ=1 without the effect of the dipole-dipole interplay. For γ>0(0→0.05), we observe when the effect of the intrinsic dissipation rate is taken in account: (i) For small *N* (N=4), the MIN, GQD, and QE have different irregular oscillations and amplitudes, and the phenomena of sudden growth and sudden death of the concurrence entanglement appear at γ/λ=0. (ii) After particular values of (λt,γ/λ) the quantifiers of MIN, GQD, and QE present stability regions in which all generated NCCs are time independent. (iii) The initial coherence intensity parameter and the intrinsic dissipation rate play an important role in the generation of NCCs.

**Figure 5:**
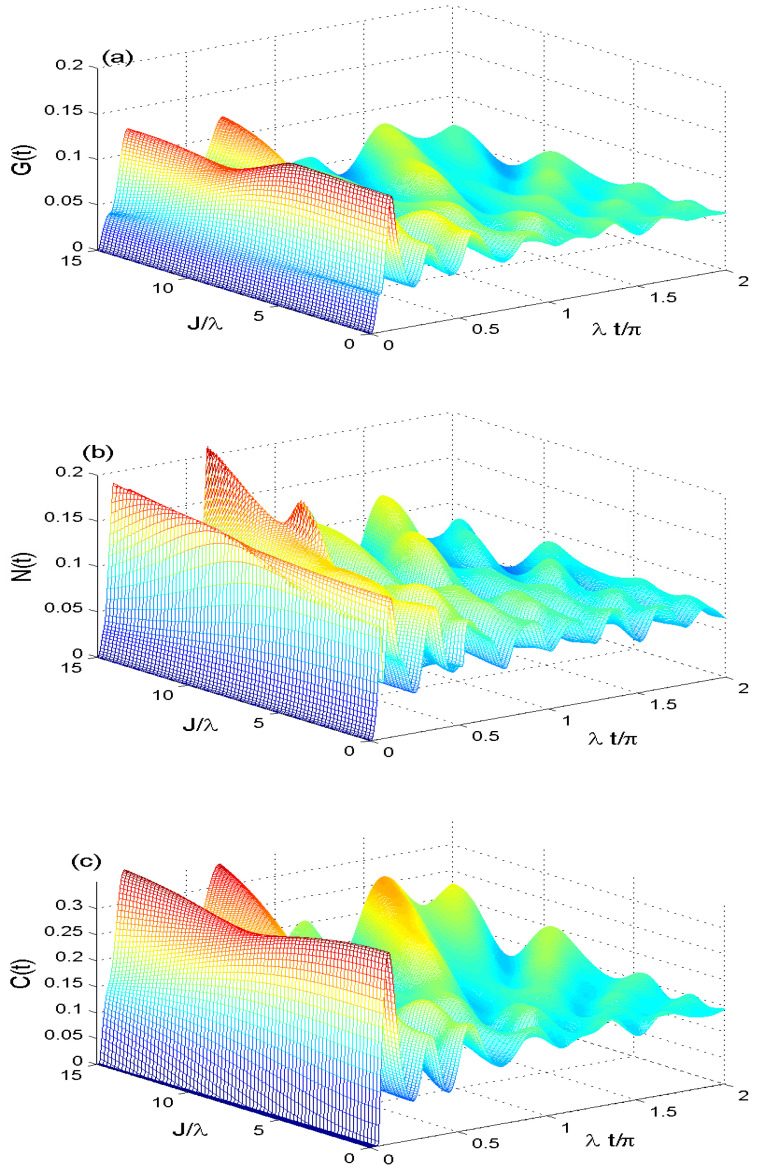
The quantifiers of G(t) in (**a**), M(t) in (**b**) and C(t) in (**c**) when J/λ∈[0,15] with smaller coherent intensity N=4, γ/λ=0.01 and κ=1.

Figure 5a–c shows the effect of the dipole-dipole interplay rate J/λ∈[0,15] on the Non-classical correlations with intrinsic dissipation rate γ=0.01, smaller coherent intensity N=4, γ/λ=0.01 and κ=1. At J/λ=0, the MIN, GQD, and QE have damped oscillations with different amplitudes due to the effect of the intrinsic dissipation. This dynamics of MIN, GQD, and QE can be changed drastically by increasing the dipole-dipole interplay rate, where the fluctuations of the NCC functions increase rapidly with a slight interference between their patterns.

The minimum and maximums values of MIN, GQD, and QE are increased under the influence of dipole-dipole interaction. It leads to increasing the irregular oscillations and disappearance of the stationary correlations. We deduce that the dipole-dipole interplay rate enhances the GQD, MIN and QE. Where the NCCs irregularity increases by increasing the dipole-dipole interplay rate.

## 5. Conclusions

We consider the model of two dipole coupled qubits placed inside a coherent cavity-field under the intrinsic dissipation rate and 2-photon transitions. The analytical solutions are derived by using the dressed states. The robustness of the generated non-classical correlations of the two qubits is investigated via different quantifiers against the intrinsic dissipation rate, dipole-dipole interplay, coherent intensity and the superposition of coherent states. The non-classical correlations can be enhanced by increasing the initial coherent intensity, and they can be frozen at their stationary correlations for a specific range of the intrinsic dissipation rate. The dynamical behavior of the nonclassical correlations depend on the system parameters. The enhancement of the dipole-dipole interplay rate leads to the increase of the geometric quantum discord and the measurement-induced non-locality as well as the entanglement. The above mentioned rate is also responsible for the inhibition of the NCCs stationarity. These results offer practical applications in the field of quantum information processing where the geometric quantum discord, the measurement-induced non-locality and the entanglement are crucial resources.
